# Preparation of Methylcellulose Film-Based CO_2_ Indicator for Monitoring the Ripeness Quality of Mango Fruit cv. Nam Dok Mai Si Thong

**DOI:** 10.3390/polym14173616

**Published:** 2022-09-01

**Authors:** Duangjai Noiwan, Panuwat Suppakul, Pornchai Rachtanapun

**Affiliations:** 1 Department of Postharvest Technology, Faculty of Engineering and Agro-Industry, Maejo University, Chiang Mai 50290, Thailand; 2 Center for Advanced Studies for Agriculture and Food (CASAF), Department of Packaging and Materials Technology, Faculty of Agro-Industry, Kasetsart University, Bangkok 10900, Thailand; 3 Department of Food Science and Biotechnology, and Center for Intelligent Agro-Food Packaging, College of Life Science and Biotechnology, Dongguk University-Seoul, Goyang-si 10326, Korea; 4 Division of Packaging Technology, School of Agro-Industry, Faculty of Agro-Industry, Chiang Mai University, Chiang Mai 50100, Thailand; 5 The Cluster of Agro Bio-Circular-Green Industry (Agro BCG), Chiang Mai University, Chiang Mai 50100, Thailand; 6 Center of Excellence in Materials Science and Technology, Chiang Mai University, Chiang Mai 50200, Thailand; 7 Postharvest Technology Innovation Center, Commission on Higher Education, Bangkok 10400, Thailand

**Keywords:** mango, pH-dye, carbon dioxide, ripening indicator, kinetic model, food packaging system

## Abstract

Day-to-day advancements in food science and technology have increased. Indicators, especially biopolymer-incorporated organic dye indicators, are useful for monitoring the ripeness quality of agricultural fruit products. In this investigation, methylcellulose films—containing pH dye-based indicators that change color depending on the carbon dioxide (CO_2_) levels—were prepared. The level of CO_2_ on the inside of the packaging container indicated the ripeness of the fruit. Changes in the CO_2_ level, caused by the ripeness metabolite during storage, altered the pH. The methylcellulose-based film contained pH-sensitive dyes (bromothymol blue and methyl red), which responded (through visible color change) to CO_2_ levels produced by ripeness metabolites formed during respiration. The indicator solution and indicator label were monitored for their response to CO_2_. In addition, a kinetic approach was used to correlate the response of the indicator label to the changes in mango ripeness. Color changes (the total color difference of a mixed pH dye-based indicator), correlated well with the CO_2_ levels in mango fruit. In the ‘Nam Dok Mai Si Thong’ mango fruit model, the indicator response correlated with respiration patterns in real-time monitoring of ripeness at various constant temperatures. Based on the storage test, the indicator labels exhibited color changes from blue, through light bright green, to yellow, when exposed to CO_2_ during storage time, confirming the minimal, half-ripe, and fully-ripe levels of mango fruit, respectively. The firmness and titratable acidity (TA) of the fruit decreased from 44.54 to 2.01 N, and 2.84 to 0.21%, respectively, whereas the soluble solid contents (SSC) increased from 10.70 to 18.26% when the fruit ripened. Overall, we believe that the application of prepared methylcellulose-based CO_2_ indicator film can be helpful in monitoring the ripeness stage, or quality of, mango and other fruits, with the naked eye, in the food packaging system.

## 1. Introduction

Mango (*Mangifera indica* Linn.) is regarded as the second-most significant fruit crop in tropical and subtropical countries. Its quality is determined by the size, shape, cultivar, taste, aroma, peel, and flesh color. Notably one of the most important cultivated varieties of Nam Dok Mai Si Thong, it is the outstanding export product in Thailand, due to its flavor and bright yellow flesh, and is exported to China, Korea, Japan, Malaysia, and Vietnam. It is produced in the north, central, and southern parts of Chiang Mai, Phitsanulok, Loei & Nakhon Ratchasima, and Prachuap Khiri Khan provinces of Thailand. Mangoes continue to ripen after harvest. Compositional change of the fruit is relevant for understanding metabolic processes [1,2,3,4,5]. Hence, postharvest handling techniques play a crucial role in the export to other countries [4,6]. This is because mangoes sold in markets are frequently at various stages of maturity. Therefore, it is critical to measure the fruit’s ripening state so that consumers can determine when it is perfect for eating.

Food packaging system-adjoined indicators provide detailed information on food’s integrity, freshness, microbial growth, temperature, and shelf life [7]. Food produce quality includes its sensory qualities, nutritional content, chemical components, mechanical characteristics, functional characteristics, and flaws [2,8]. Because buyers view ripeness as the primary quality indicator, monitoring and regulating it is becoming a crucial issue in fruit management. To assess fruit quality and ripeness quality, soluble solids concentration, titratable acidity, flesh firmness, and peel color are typically used. [2,9]. Generally, most of them require the destruction of the samples used for analysis. To assess fruit quality, numerous non-destructive methods have been developed. When fruit is being stored, non-destructive electronic nose technology can be used to assess its state of ripeness [10]. Lebrun et al. [11] attempted to differentiate between mango fruit maturity by employing FOX 4000 e-nose, and GC. When separating the fruit from different harvest maturities of green and ripe fruit, the e-nose showed better results than the GC testing. The e-nose could be a useful and rapid device with which to sort peaches into three categories: unripe, ripe, and over-ripe. The techniques have been used to assess the chemical and physical characteristics of pear from their e-nose signal [12,13,14]. However, Gomez et al. [15,16] reported that the association between the measured and anticipated values of fruit (tomato and mandarin) revealed a very low to reasonable prediction, using e-nose signals. The fruit ripeness stage was evaluated by firmness, which decreases as they ripen. Ultrasound is an acoustic method used, particularly, in the assessment of fruit quality. It has been demonstrated that variations in ultrasonic attenuation correspond to changes related to the fruit’s physiological and chemical composition. Using ultrasonic technology, mango fruit’s postharvest shelf-life has been measured [17]. Mizrach [18] measured the attenuation of the mango fruit’s ultrasonic signal after 10 days at room temperature storage and found that it increased from 2.7 dB/mm on the first day to 4.16 dB/mm at the end of the test. Vergara et al. [19] described the development of a radio frequency identification (RFID) reader, embedded with micro-machined metal oxide gas sensors, for monitoring climacteric fruit during shipping and vending. The reader can work as an alarm-level monitor, identifying abnormal ethylene levels or acetaldehyde and ethanol. Recently, imaging techniques have been used for monitoring fruit ripening and maturity [20]. The advancement of non-destructive technologies for determining fruit ripening stage has been the focus of all research, and these techniques have several benefits [21]. 

However, these ripeness indicator methods have been utilized to forecast fruit quality features that are frequently identified by conventional destructive techniques (SSC, TA, firmness, etc.). Therefore, there is still a need for a simple, accessible, and economical method. A ripeness indicator potentially offers a small, low-cost, and user-friendly device for monitoring fruit quality, which is used to indicate the fruit ripening state. The amount of published work on this subject is still limited. One alternate suggestion to meet this requirement is the development of intelligent packaging, in the form of a food quality indicator, to monitor freshness status. The term "intelligent packaging" refers to a type of packaging that can perform intelligent tasks to aid in decision-making for improving quality, extending shelf life, and providing information and warnings about potential issues [22]. Generally, the pH, temperature, and storage time play crucial roles in fruit ripening. For intelligent packaging, polymer-based indicators are used for food quality, fermentation, and ripeness, because of their hydrophilic, better adhesive, and non-toxic nature. The colorimetric indicator films are fabricated using various biopolymers (cellulose, starch, chitosan) with the pH dyes (bromophenol blue, chlorophenol red, methyl red, molybdenum blue). These indicators change color when reacting with CO_2_, ethylene, and volatile organic compounds [23,24,25,26]. These metabolite derivatives have been produced by agricultural fruits and products during the storage periods. The indicator color changes help to determine the quality of ripeness and spoilage of agricultural fruits and products. Kuswandi et al. investigated cellulose membrane–bromophenol blue-based indicator to determine the *Psidium guajava* freshness state. The indicator changed from blue to green with acetic acid, confirming the ripening [24]. Kuswandi and Murdyaningsih reported a chlorophenol red membrane package indicator label for grapes during storage time. The label reacted with reduced volatile organic acid, changing color from white to beige to yellow, due to the ripening stages [23]. Generally, biopolymers such as methylcellulose [27] are tough, non-toxic, odorless, and provide better adhesive properties. The fermentation and ripening stage is determined from the level of CO_2_, using the polymer-based poly(ether-block-amide) indicator [7,28]. A ripeness indicator is a type of intelligent packaging that employs metabolites as "information" with which to track the ripening of fruit. It can either be a packaging system or a material [29]. The goal of this work is to create a methylcellulose film containing a pH dye-based ripeness indicator for monitoring the ripening of mango fruit cv Dok Mai Si Thong, Nam Dok. 

## 2. Materials and Methods

### 2.1. Chemicals

Food-grade methylcellulose (MC) (Methocel®, Dow Chemical, New York, NY, USA) was used as the carbohydrate biopolymer for the label base. Polyethylene glycol-400 (Chemipan Corporation Co., Ltd. Bangkok, Thailand), Bromothymol blue (Ajax Finechem, Auckland, Australia), and ethyl alcohol (Merck, Darmstadt, Germany) were used to prepare a dye mixture. Acetic acid (Merck, Darmstadt, Germany) was used to evaluate the sensitivity of dye mixtures. 

### 2.2. Ripeness Indicator Fabrication 

#### 2.2.1. Preparation of Color Indicator Solution

A method was modified from Nopwinyuwong et al. [25,30]. The indicator solution was made by combining bromothymol blue (0.04%, *w*/*v*) in ethanol (50%, *v*/*v*) and methyl red (0.04%, *w*/*v*) in ethanol (50%, *v*/*v*) in a ratio of 3.8:1.2, which is a concentration of 1% (*v*/*v*) in ethanol (50 %, *v*/*v*).

#### 2.2.2. Preparation of the Color Indicator Label

The coating solution was prepared by dissolving methylcellulose (3%, *w*/*v*) in 50% aqueous ethanol solution. Polyethylene glycol-400 (1%, *w*/*v*) was used as a plasticizer and this mixture was then homogenized at 22,000 rpm until complete dissolution. This solution was used to produce an indicator label by adding pH dye solution at a 3.8:1.2 ratio. Further, the film-forming solutions were applied to polyethylene film and allowed to dry for 24 h at ambient temperature (25 °C). The obtained ripeness indicator was conditioned at 25 °C and 65% relative humidity.

### 2.3. Sensitivity of Mixed pH Dyeing Indicator

#### 2.3.1. Measurement of Mixed pH Dyeing Solution Sensitivity

The color change of the mixed pH- dyeing solution was observed by testing with 0.1 M acetic acid. The test tube contained 10 ml of mixed pH-dyeing solution, and then different concentrations (30, 35, 40, 45, 50, 55, 60, 65, 70, 75, and 80 µL) of 0.1 M acetic acid were added. The pH value and absorbance of solutions were measured with a pH meter and spectrophotometer (at 416 nm), respectively.

#### 2.3.2. Color Changes in Indicator Labels Caused by Carbon Dioxide (CO_2_)

The ripeness indicator label was tested with carbon dioxide as a metabolite compound, known as the accurate weight. CO_2_ was diluted with nitrogen and injected into the glass jar with a gas-tight syringe, obtaining CO_2_ concentrations of 0–3.0 % (*v*/*v*). The color change in the label indicator was evaluated with a color meter (Minolta Model CR-400, Japan) and expressed as Hunter system [31].

### 2.4. Applications of Ripeness Indicator Label with “Nam Dok Mai Si Thong” Mangoes

#### 2.4.1. Experiment Setup

Mangoes were harvested at commercial maturity and transported to the laboratory immediately. Mangoes of uniform size (320–350 g), and free from defects, were selected and placed into 800 mL plastic boxes. Each plastic container was attached to an indicator label, and the samples were kept at 13, 20, 27, and 34 °C. The quality changes of fruit stored at different temperatures were assessed, and this was compared to the color changes in the label indicators.

#### 2.4.2. Quality Changes during the Fruits’ Ripening Period

Flesh firmness was measured by Noiwan et al. [4]. The soluble solid content (SSC) was determined using the method of Sivakumar et al. [32]. The titratable acidity (TA) was conducted by the addition of 0.01 N NaOH to pH 8.1 [33].

#### 2.4.3. Kinetics Study of Indicator Labels 

The color changes in indicator labels that were attached inside the plastic boxes were measured with a color meter and expressed as total color difference (∆*E*). The ∆*E* was calculated according to the following equations:

∆*E* = [(Δ*L∗*)^2^ + (Δ*a∗*)^2^ + (Δ*b∗*)^2^]^1/2^ (1)

where Δ*L∗* is the difference in brightness between day zero and each time period.

The redness–greenness difference between day zero and each time period is represented by Δ*a∗*.

The yellow-blue difference between day zero and each time period is represented by Δ*b∗*.

By fitting a zero-order Equation (2), a first-order Equation (3), a second-order kinetic model Equation (4), or the following [34] Equation (5) to the experimental data, the kinetics of indicator labels were explained:

*F*(*X*) = [*X*_0_] − [*X_t_*] (2)

*F*(*X*) = ln [*X*_0_ *− X_t_*] (3)

(4) $$F(X)=\frac{1}{X_{0}}\;-\;\frac{1}{X_{t}}$$

(5) $$F(X)=\sqrt{\ln\;(\frac{1}{1\;-\;X})}$$

where [*X*_0_] represents the initial values of total color difference (TCD) and [*X_t_*] represents the values of TCD for a specified storage period and temperature. The values of TCD were measured using the corresponding value *X* as the dynamic parameter, and the relationship was described in terms of the response function, as follows:

*F*(*X*) = *kt* (6)

where *X* represents the values of: ∆*E*; *k:* the reaction’s temperature-dependent rate constant; and *t:* the amount of time it has been stored. A straight line may be drawn by graphing a curve between the total color difference response function *F(X),* and time, and the slope used to determine the *k* of various storage temperatures. Taking the natural logarithm on both sides of the Arrhenius function:

ln*k* = ln*A* + *E_a_*/*RT* (7)

by plotting a curve between ln *k* and 1/*T*, a straight line was obtained. The activation energy could be calculated from the slope, and *A* from the intercept, directly. 

### 2.5. Statistical Analysis

The experiment followed a completely randomized design of three replicates per treatment (*n* = 3). SPSS was used to examine the data (Version 11). A one-way analysis of variance (ANOVA) was performed using Duncan’s multiple range test (*p* ≤ 0.05).

## 3. Results and Discussion

### 3.1. Color Change of Indicator Solution

**Figure 1:**
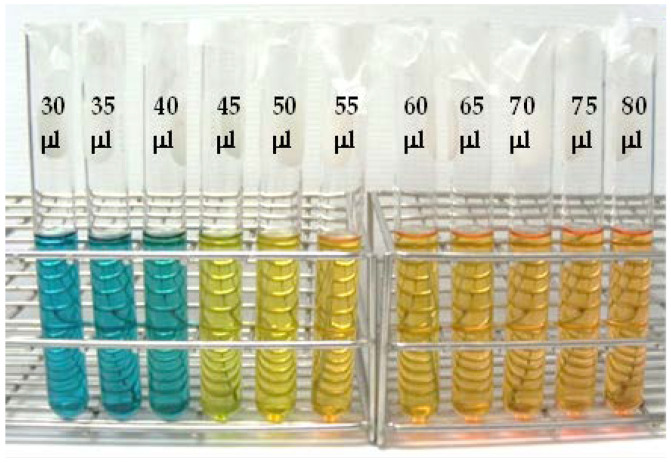
Colorimetric changes of indicator solution with addition of different concentrations (30, 35, 40, 45, 50, 55, 60, 65, 70, 75, and 80 µL) of 0.1 M acetic acid.

Different concentrations (30, 35, 40, 45, 50, 55, 60, 65, 70, 75, and 80 µL) of 0.1 M acetic acid were added to the vials containing colorimetric mixed pH dye-based indicator solution, causing a visual color change of the solution from blue to yellow (Figure 1). As a result, the pH decreased from 10.59 to 5.91 (Table 1). The colorimetric changes were determined by absorption at 416 nm. Initially, the absorption increased, and the maximum value was observed at 45 µL concentration, then the absorption significantly decreased. 

### 3.2. Change in Color of Color Indicator Label

**Figure 2:**
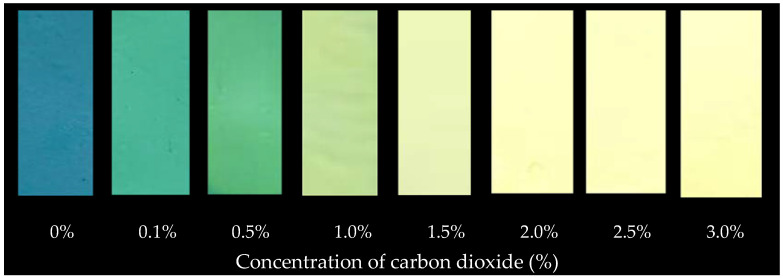
Change in color of indicator label in response to CO_2_.

The color of the indicator label was measured using a CIE L*, a*, and b* scale, when contrasted with CO_2_, where L* indicates lightness, a* indicates chromaticity on a green (−) to red (+) axis, and b* indicates chromaticity on a blue (−) to yellow (+) axis. Lightness (L*) value increased because the indicator label changed from blue, to light green, to yellow, similar to the a* value, which increased (Table 2). The various color changes of colorimetric indicator labels, as shown in Figure 2: when exposed to CO_2_ levels between 0 and 3.0 percent (*v*/*v*), indicator labels were found to exhibit, correspondingly, a clear spectrum from blue to light green to yellow. To simply and accurately track the degree of fruit ripening of packaged mango fruit in a non-destructive manner during distribution and retail sale, colorimetric mixed pH dye-based markers are being applied to mango fruit packaging. The levels of CO_2_% correlated with the L*, a*, and b* values of the indicator label, helping to determine the Mango fruit ripening stage during storage at various temperatures.

### 3.3. Color Changes and Kinetics Study of Indicator Labels during Mango Ripening 

In packed mango fruits, headspace gas that has been dissolved in the hydrophilic material of the filter layer produces carbonic acid when moisture is present. Diprotic carbonic acid, pKa: 6.36 at 25 °C [35], has two hydrogen atoms that have the potential to separate from the parent molecule to create hydrogen ions (H^+^) and bicarbonate ions (HCO_3_^−^). A hydrogen ion then joins with a water molecule, as a proton, to create a hydronium ion (H_3_O^+^). Hydronium ions interact with the indicator label’s basic form (In), creating an acid form (HIn), which causes the indicator label to change color. [7,36,37]:

CO_2_(g) ↔ CO_2_ (aq)

CO_2_ (aq) + H_2_O ↔ H_2_CO_3_

H_2_CO_3_ ↔ HCO_3_‾ + H^+^

H^+^ + H_2_O → H_3_O^+^

H^+^ + In‾ → HIn

**Figure 3:**
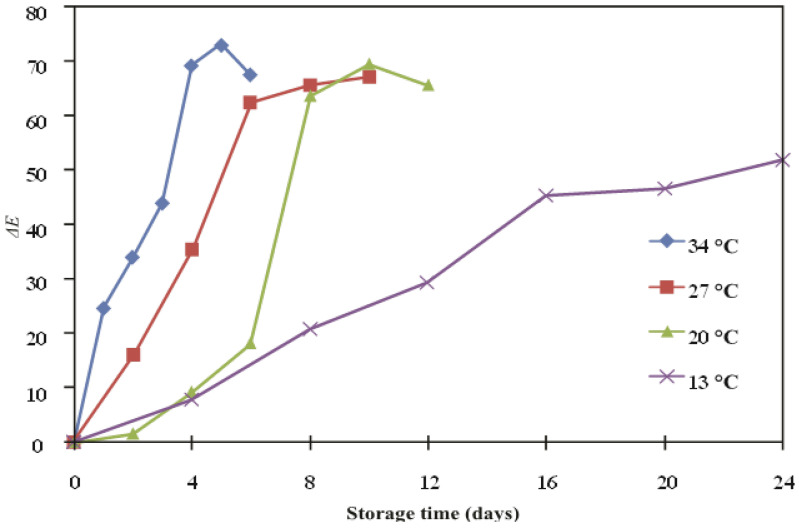
Effect of storage time and temperatures on ∆*E* values of indicator labels in the packaged mangoes.

The indicator labels’ response caused a constant and consistent shift in the ∆*E* value as well. With time, ∆*E* values increased. The indicator labels’ final ∆*E* values ranged from 51 to 73, as shown in Figure 3. Based on the observation of this data, 27 °C storage, up to day 6, leads to better ∆*E* values when compared to other mango storage temperatures. It is generally accepted that ∆*E* values larger than 5.0 are plainly discernible to the naked eye and that ∆*E* values greater than 12.0 denote an entirely separate color space [38]. Even though they occurred at a distinct rate, the variations in the label of ∆*E* exhibited a Gaussian kinetic model [Equation (5)] [34]. Therefore, the observable ∆*E* change of the indicator label can be used as a model for kinetic technique. 

**Figure 4:**
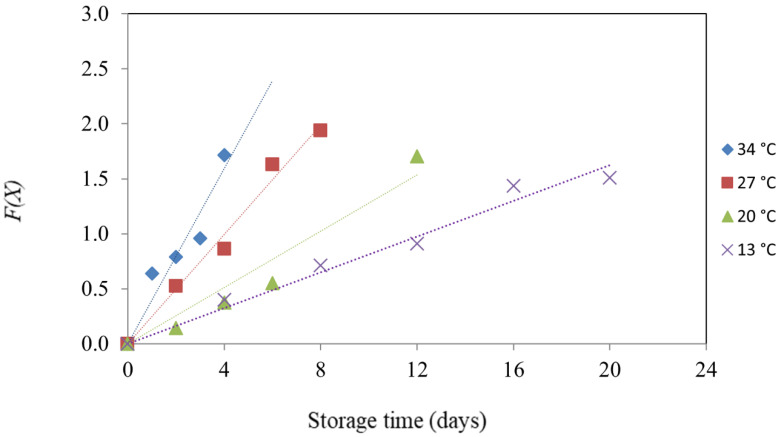
Plot of the response function *F*(*X*) with storage time for ∆*E* value of indicator label applied with mango fruit at different temperatures.

Figure 4 illustrates what happens when the function *F(X)* of ∆*E* is plotted against storage time. Further, it shows four straight lines with various slopes at various temperatures. Table 3 lists the calculated kinetic parameters as rate constants (*k*) and coefficients of determination (*R*^2^). The *R*^2^ of each function parameter was adequately high (between 0.9150 and 0.9845, as shown in Table 3), demonstrating the model’s suitability. The Arrhenius equation [Equation (7)] was used to demonstrate that the temperature dependency was explained with the ∆*E* values, due to the temperature dependence. Based on the changes in ∆*E* of the indicator label during the mango fruit’s ripening, the values of *E_a_* as 52.30 kJ/mol could be estimated (Table 3). The mango fruit’s firmness, SSC, and TA value all serve as ripening indicators. The values of *E_a_* were 46.45, 43.05, and 54.22 kJ/mol, respectively, which changes the firmness, SSC, and TA of the mango fruit during ripening [4]. The indicator is used to accurately track the degree of fruit ripening, if it has a similar temperature dependence to the processes that lead to product quality decline, which translates into activation energies that differ by less than 25 kJ/mol. According to research on the kinetics of fresh produce, based on the Arrhenius equation, the activation energy of carbon dioxide, generated during golden drop spoiling, is 48.98 kJ/mol, and the overall color difference of freshness-indicator labels results in activation energy of 49.25 kJ/mol. The indicator response in samples of golden drops was discovered to be correlated with microbial growth patterns, allowing for the real-time monitoring of deterioration [30]. Rukchon et al. [39] developed the food spoilage indicator for monitoring the freshness of skinless chicken breast. The kinetic approach correlates the reaction of the indicator label with variations in skinless chicken breast spoilage. The overall color difference of a mixed pH dye-based indicator, which measures color variations, correlated well with the CO_2_ levels of skinless chicken breast.

**Figure 5:**
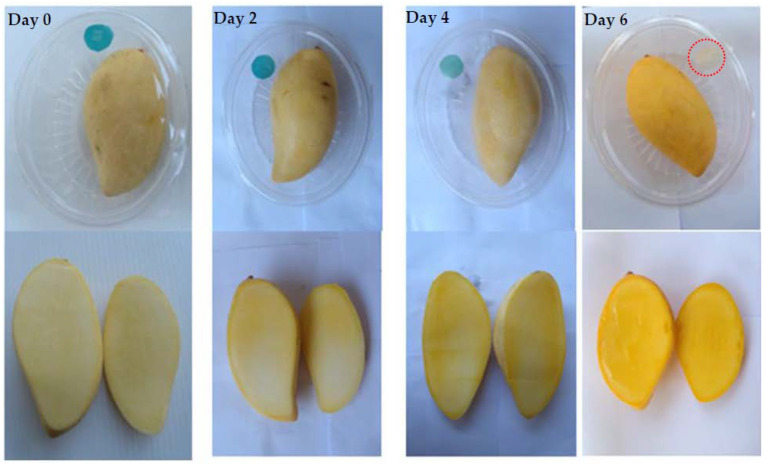
Color change of ripeness-indicator label with packaged mango fruit cv. Nam Dok Mai Si Thong at 27 °C; blue = unripe, light green = half-ripe and yellow = fully-ripe.

Recently, Perez et al. reported the ripening process, during storage, in sapote mamey fruit, showing that higher temperatures in the storage periods induced the more active softening enzymes. Further, low temperature (10 °C) created little change in the firmness of the fruit, and 20–25 °C temperature was considered suitable in softened fruit [40]. Figure 5 illustrates the shift in colorimetric mixed pH dye-based ripeness indicator labels on packaged mango fruit at 27 °C. These results demonstrate that, during storage at 27 °C, the minimal, half-ripe, and fully-ripe levels of mango fruit occurred on days 2, 4, and 6, respectively. When exposed to CO_2_ during storage, indicator labels displayed a distinct spectrum from blue to light bright green to yellow. According to the mango fruit’s hardness, SSC, and TA values, which serve as ripening indices, the obtained results revealed that the firmness and TA of fruits declined, whereas the SSC of fruits increased, as shown in Table 4. Similarly, Noiwan et al. [4], reported that over 6 days of ‘Nam Dok Mai Si Thong’ mango fruit storage, the firmness decreased, and all were most stable up to day 10. In the mango, on storage day 6, the SSC, and TA reached almost the maximum level. Carbon dioxide metabolite was emitted from mango fruit during ripening. Previous studies revealed that the ripening metabolites of mango produced CO_2_, malic and citric acids. During storage, the CO_2_ levels also increased as the days increased. Obtained results show that the incremental increase of CO_2_ levels is related to the temperature and activation energy, which changes the mango flesh color, taste, and texture during ripening [4,28,41]. Recently, Baek et al. [7,28] investigated the poly(ether-block-amide) film–based CO_2_ indicator that has been used for monitoring the quality of packaged kimchi during storage. Polymer-based indicators offer an improved understanding of ripening processes, whereas further advancements are required for improved commercial indicators useful in the food-packaging industries.

## 4. Conclusions

The ripeness indicator was developed in this study to monitor the ripeness quality of mango by CO_2_ concentrations in the headspace of mango packages. The Gaussian kinetic model and Arrhenius equation, used to evaluate the levels of mango fruit ripening via the formation of CO_2_, were correlated to the ∆*E* values of each indicator label. A kinetic model was well-fitted with a total color difference *(*∆*E*) during storage. The indicator response was found to correlate with carbon dioxide released in mango fruit. During fruit ripening periods of 0, 2, 4, and 6-day storage at 27°C, the prepared methylcellulose film containing mixed pH dye-based indicator reacted with different levels of CO_2_ and indicated the three different real-time monitoring-of-ripeness stages: blue in the minimal ripening; light bright green during half-ripening; and yellow at full ripeness. These color changes are evaluated by the quality index of ripeness of mango. During storage days 0~6, mango firmness was drastically reduced from 44.54 to 2.01 N. Due to the ripening process of mango, the hard texture changed to soft, and the flesh color changed from light yellow to dark yellow. In addition, the total acidity was decreased, which produced an impact on flavor as well as pH. The physiological process of respiration in the mango led to the creation of simple sugars, having an effect on the maturity and taste of the fruit. Therefore, The SSC increased from 10.70 to 18.26% when the fruit ripened. The ripeness indicator label could be a useful and rapid device for showing the ripening stages of mango fruit cv. Nam Dok Mai Si Thong. However, we hope that this slight advancement of the methylcellulose indicator label will be helpful in allowing consumers to easily monitor, with the naked eye, the ripening quality and shelf life of the mango.

## Figures and Tables

**Table 1:** Change in pH and Absorbance of indicator solution in response to acetic acid.

0.1 M Acetic Acid (µL)	pH	Absorbance (416 nm)
30	10.59	1.4191
35	10.04	1.5552
40	9.04	1.6053
45	8.1	1.6082
50	7.64	1.6065
55	7.04	1.6009
60	6.66	1.5987
65	6.35	1.5765
70	6.17	1.5624
75	6.01	1.5616
80	5.91	1.5524

**Table 2:** Change in L* a* and b* value of indicator label in response to CO_2_.

Carbon Dioxide (%)	L*	a*	b*
0	40.38	−16.3	−19.14
0.1	41.52	−21.9	−6.18
0.5	49.34	−25.74	−4.57
1	58.09	−22.38	10.18
1.5	62.72	−18.22	17.28
2	70.84	−8.34	26.78
2.5	74.93	−3.81	29.6
3	78.41	−0.28	34.09

**Table 3:** Response of rate constants (*k*), the correlation coefficient (*R*^2^) of the fit and activation energy (*E_a_*) obtained from the ∆*E* model.

Temperature (°C)	∆*E*		
	k (day^−1^)	R^2^	*E_a_* (kJ/mol)
13	0.77	0.9737	52.3
20	0.16	0.9417	
27	0.249	0.9845	
34	0.271	0.915	

**Table 4:** Effect of changes in mango fruit cv. Nam Dok Mai Si Thong firmness, SSC, and TA during storage at 27 °C.

Storage Time (Days)	0	2	4	6
SSC (%)	10.70	11.78	14.82	18.26
TA (%)	2.84	2.38	1.29	0.21
firmness (*N*)	44.54	12.94	3.44	2.01

## Data Availability

The data presented in this study are available on request from the corresponding author.
